# Supplementary material for: Unveiling the radiative local density of optical states of a plasmonic nanocavity by STM
Source: Nat Commun. 2020 Feb 24;11:1021. doi: 10.1038/s41467-020-14827-7 (PMC7039974; doi:10.1038/s41467-020-14827-7)
Supplement: Supplementary file 1 — Supplementary Information [file 41467_2020_14827_MOESM1_ESM.pdf]

Supplementary Information: Unveiling the Radiative Local Density of Optical States of a Plasmonic Nanocavity by STM

Alberto Martín-Jiménez[1], Antonio I. Fernández-Domínguez[2], Koen Lauwaet[1], Daniel Granados[1], Rodolfo Miranda[1,3], Francisco J. García-Vidal[2,4*] & Roberto Otero[1,3*]

[1]IMDEA Nanociencia, Madrid, Spain

[2]Departamento de Física Teórica de la Materia Condensada and Condensed Mater Physics Center (IFIMAC), Universidad Autónoma de Madrid, Madrid, Spain

[3]Departamento de Física de la Materia Condensada and Condensed Mater Physics Center (IFIMAC), Universidad Autónoma de Madrid, Madrid, Spain

[4]Donostia International Physics Center (DIPC), E-20018 Donostia-San Sebastián, Spain

E-mail: fj.garcia@uam.es, roberto.otero@uam.es

Supplementary Note 1: Robustness of the fitting procedure

In order to characterize accurately the shifts of the high- and low-energy plasmonic modes, we have fitted our curves to a series of Gaussian curves. While the spectra show two prominent peaks, in good agreement with the theoretical calculations, they also show some further structure in the low energy region. We have found that the minimum set of peaks that yield a reasonable overall fit of the experimental spectra is four, and all the analysis shown in this communication has been done following this fit. To ascertain the possible role of our choice fitting function on the peak positions, we have also performed a fitting with five Gaussian functions. As expected, since the parameter space is larger, the fitting is better with five peaks (see Supplementary Figure 1 for a comparison between the fittings using five (a) and four (b) Gaussian curves on the spectra recorded with V_{Bias}^{st}=3.4 V, where χ^2 is reduced from 0.0683 to 0.0574), and in particular, the low energy structure is slightly better reproduced. The effect on

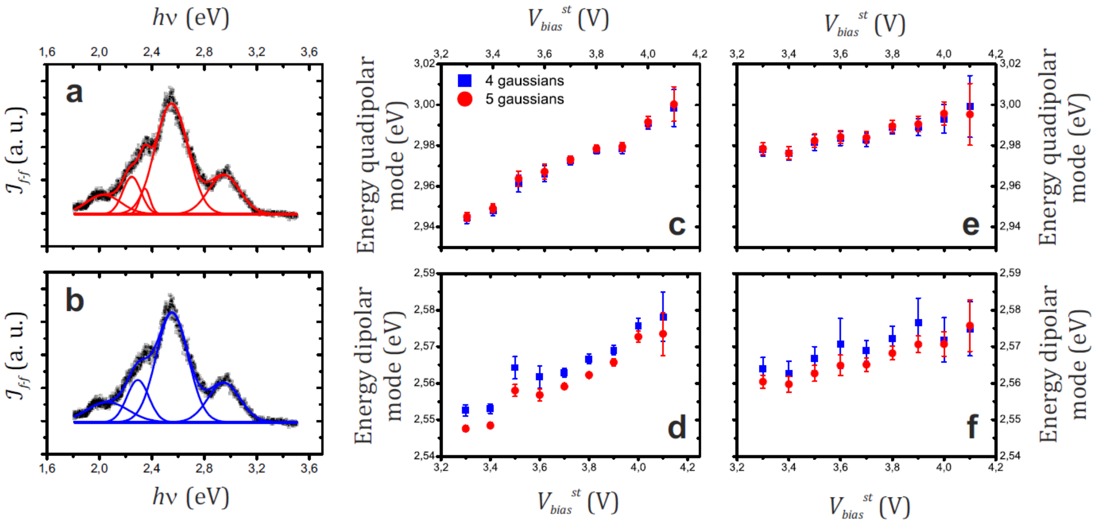

Supplementary Figure 1. Raw spectra recorded with a bias voltage of 3.4 V and fitted with either five (a) or four (4) Gaussian peaks. c) and d) Peak positions for the high- and low-energy plasmonic modes of different stabilization voltages extracted from a fitting of the raw spectra using four (blue squares) and five (red dots) Gaussian curves. e) and f) Peak positions for the high- and low-energy plasmonic modes of different stabilization voltages extracted from a fitting of the normalized spectra using four (blue squares) and five (red dots) Gaussian curves. Error bars in panels c-f correspond to the standard deviation obtained from the Gaussian fitting.

the position of the main peaks is however small. Supplementary Figure 1 displays the peak position for the quadrupolar (c) and dipolar (d) mode energies in the raw spectra fitted with four and five Gaussians. While the energy of the quadrupolar mode is not affected at all by the inclusion of a new peak, the dipolar energies are systematically reduced by only 3-5 meV. Moreover, the shifts are not affected by the choice of fitting function within the error of the method. Similar results are obtained when comparing the normalized spectra (panels (e) and (f)). We thus conclude that the results described in this paper are rather insensitive to the precise choice of the fitting function, highlighting the robustness of our method to extract energy shifts from the experimental data.

Supplementary Note 2: Estimation of the tip-surface distance (cavity gap size)

Due to the relatively large voltages required for the excitation of the plasmonic modes, the $I(V)$ curves are no longer well-described by a linear dependence in all the voltage range. Thus, relating directly the total tunnelling current at each stabilization voltage with the tip-surface

distance according to the approximate expression $I_t = VG_0 e^{-2\delta\sqrt{2m/_{\hbar^2}(\phi_t+\phi_s)/_2}}$ can no longer be expected to yield reasonable values[1]. Here we follow a slightly different approach based on the limit of Equation (2) for very low temperatures, so that the Fermi functions can be taken as Heaviside step functions. Under this conditions we retrieve the well know expression

$$I_t \propto \int_0^{eV} \rho_t(E - eV)\rho_S(E)T_{el}(E, V, \delta)dE \quad (1)$$

Thus, it can be easily checked out that the zero-bias conductance is given by

$$\left.\frac{dI}{dV}\right|_{V=0} = \rho_t(E_F)\rho_S(E_F)T_{el}(E_F, 0, \delta) \quad (2)$$

This expression is only valid at zero bias, since, for any other bias, the derivatives of the density of states and the transmission factor with the bias should be taken into consideration. The interesting feature of Equation S2 is that the only way in which it can depend on the stabilization bias is through the modification of the tip sample distance δ. These values can then be extracted from the zero-bias conductance according to the expression

$$\left.\frac{dI}{dV}\right|_{V=0} = G_0 e^{-2\delta\sqrt{2m/_{\hbar^2}(\phi_t+\phi_s)/_2}} \quad (3)$$

where G_0 is a distance independent prefactor which we take to be equal to the conductance quantum. The result of such analysis is shown in Supplementary Figure 2. Panel a) displays the evolution of the $I(V)$ curves for different stabilization voltages. The inset demonstrates that they are reasonably linear up to about 150 meV, with a slope that decreases with increasing stabilization voltage. From our previous discussion, the decrease in the zero-bias conductance must be related to the increased tip-surface distances for increasing stabilization voltages.

The distances for each stabilization voltage are shown in Supplementary Figure 2b. The obtained values are plausible, between 1 and 1.4 nm for the relatively soft tunnelling conditions used in the experiment (0.47 nA, 2.6-4.1 V). The difference between the smallest and the largest gap sizes for these tunnelling conditions is of about 0.35 nm, and is

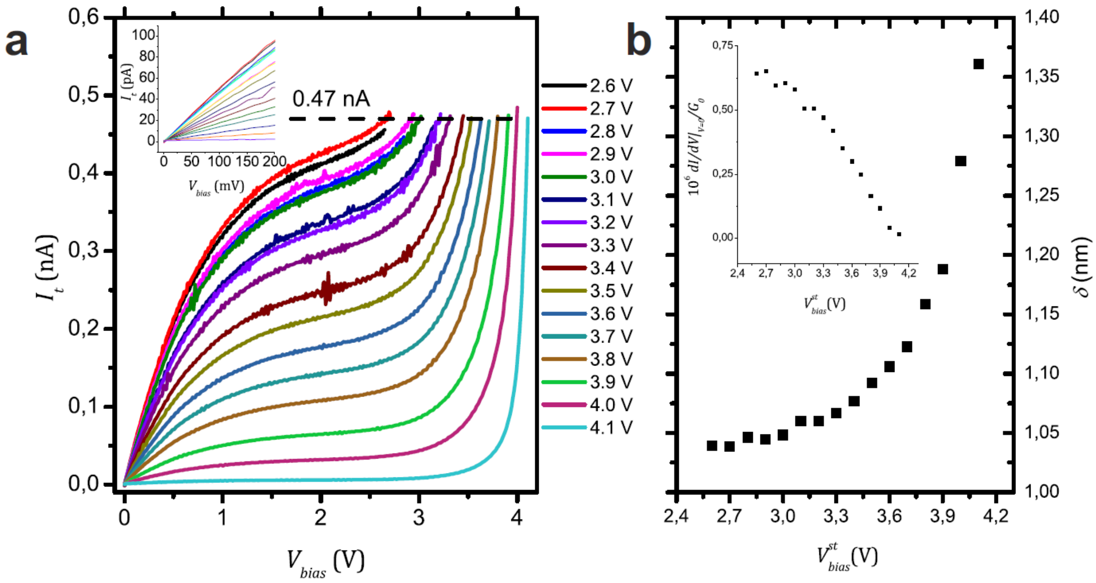

Supplementary Figure 2. a) Evolution of the $I(V)$ curves and the zero-bias conductance for different stabilization voltages. b) Tip-surface distances extracted from the analysis described in the text.

independent of the prefactor chosen in Supplementary Equation 3. The absolute distances, however, do depend on the choice of the prefactor and should thus be taken with care, since this prefactor need not be precisely the quantum of conductance G_0. However, and because of the fact that the obtained values of δ depend only logarithmically on the prefactor, to have values that differ significantly from those reported in Supplementary Figure 3, the prefactor must change by orders of magnitude. For instance, for the gap size range to shift from 1-1.4 nm to 0.5-0.9 nm, the prefactor should be lower than the quantum of conductance by more than two orders of magnitude, which is unlikely.

Supplementary Note 3: Theoretical Far-field spectra.

In this section, we present numerical calculations exploring the validity of the particle-on-a-mirror electromagnetic model of the STM tip nanocavity introduced in the main text and sketched in Supplementary Figure 3a. The structure consists of a gold sphere on top of a silver flat substrate, excited by a dipole-point-source situated at the gap center. The near-field PhDOS is obtained by integrating the time-averaged Poynting vector across a closed surface located inside the gap and surrounding the source[2]. Mimicking the experimental setup, the far-

field spectra result from the integration of the time-averaged Poynting vector across a solid angle similar to the one covered by the detector, 20° above the flat silver surface. In the simulations, this is placed up to 20 microns away from the nanocavity.

Supplementary Figure 3b plots the spectral position of the dipole and quadrupole plasmonic modes versus the gap size (the inset includes the spectra used to extract the energies of the far-field maxima). Finally, Supplementary Figure 3c renders the dipolar frequency versus the quadrupolar one, in a similar way as in Figures 1d and 5d. We can observe a linear dependence between the shifts experienced by both plasmonic modes down to 0.5 nm, where the slope is significantly increased. According to recent literature[3,4], in this gap regime quantum tunnelling and nonlocal effects, not considered in our model, become relevant. Thus, we conclude that our local calculations overestimate the red-shifting experienced by plasmon resonances as tip and substrate are approached below $\delta = 0.5$ nm. We attribute the slope change in Supplementary Figure 3c to this inherent limitation of our model.

Supplementary Figure 4 analyses the influence of the nanoparticle shape on the STML cavity far-field spectrum and PhDOS. The left panel plots normalized $\mathcal{I}_{f-f}$ versus frequency for prolate and oblate spheroidal tips. Taking as reference the 5 nm radius nanosphere (blue line, also shown in Fig. 2 in the main text), we explore the effect of increasing the tip semi-axes within the horizontal plane, R_1, or along the vertical direction, R_2. For prolate geometries, in which R_1=5 nm and R_1/R_2 is decreased down to 0.5, the far-field spectral weight of the dipolar gap plasmon peak

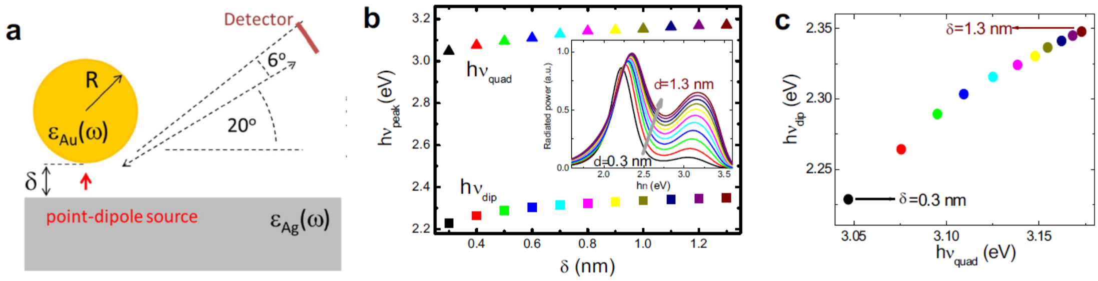

Supplementary Figure 3. Far-field spectrum calculations. **a** Sketch of the geometry considered in the theoretical modelling. **b** Plasmon energies versus gap size (retrieved from the spectra in the inset). **c** Dipolar versus quadrupole frequencies for the tip-substrate configurations in panel **b**.

(low frequency) grows significantly. This trend is accompanied by a slight redshift experienced by this peak. On the contrary, by increasing R_1/R_2 up to 2 setting R_2=5 nm, we find that the response of oblate tips remains very similar to the spherical one. The relative height of both dipolar and quadrupolar maxima is not altered, although both are slightly reduced and redshifted. These results suggest that R_2 (the semi-axis along the vertical dimension) is the key parameter establishing the general profile of the far-field spectrum of the nanocavity.

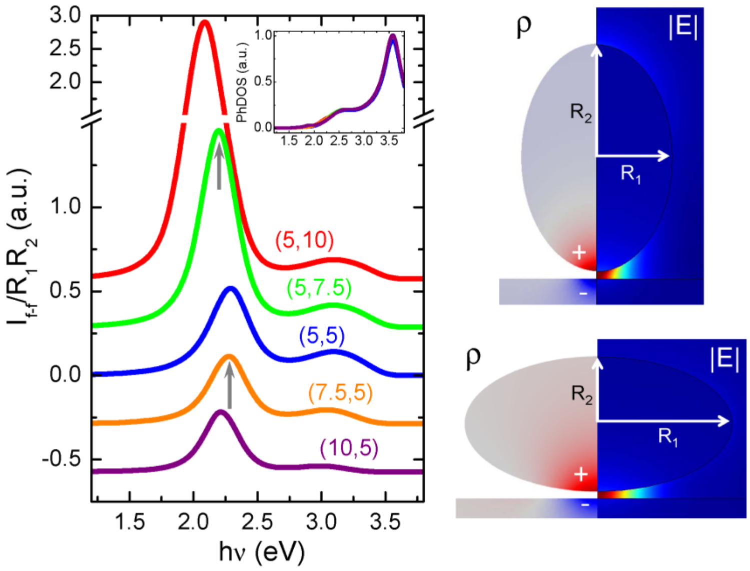

Supplementary Figure 4. Far-field spectra for prolate (top) and oblate (bottom) spheroid nanocavities. Each line is labelled as (R_1,R_2) in nm, the ellipsoid semi-axes within the horizontal plane and along the vertical direction, respectively. The inset shows the total PhDOS for the five geometries in the main panel, which overlap almost perfectly. The right panels show the induced charge and electric field amplitude maps for 1.5× prolate (top, green line) and 1.5× oblate (bottom, orange line) tips at hν~2.0 eV (see grey vertical arrows).

The inset of the right panel in Supplementary Figure 4 renders the total PhDOS for the structures in the main panel. They overlap perfectly, as they are governed by high-frequency, strongly confined, dark plasmonic modes. These, contrary to their bright counterparts, are not sensitive to the geometric deformations introduced in the tip shape. The left panels in Supplementary Figure 4 display induced charge and electric field amplitude maps for prolate (top) and oblate (bottom) spheroidal cavities with R_1/R_2 equal to 2/3 (green) and 3/2 (orange) respectively. All maps are evaluated at the dipolar plasmon resonant frequency (~2 eV, see grey arrows). These

contourplots show that the resonant electric fields associated with dipolar gap plasmons are less localized in oblate tips than in prolate ones.

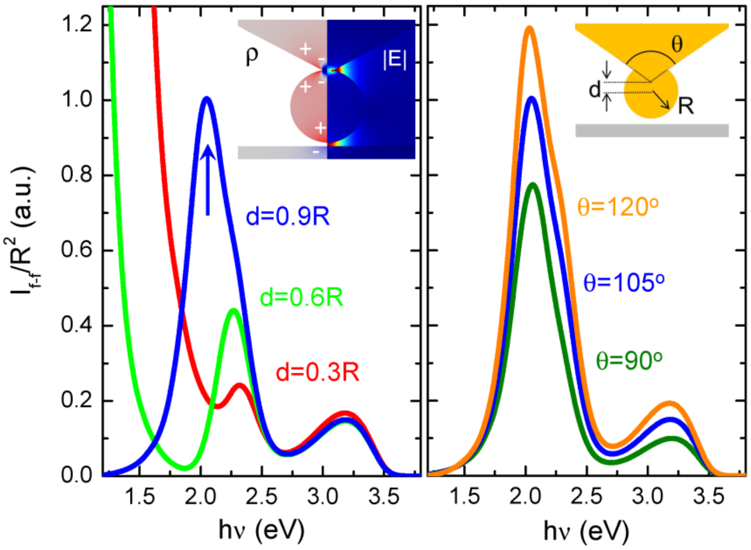

Supplementary Figure 5: Far-field spectra for conical tips decorated with an spherical (R=5 nm) protrusion at their apex. Two parameters characterize the tip geometry: θ and d (see right inset). In the right panel, θ=105°, in the right panel, d=0.9R. The left inset shows the induced charge and electric field amplitude maps evaluated at hν=2 eV, θ=105° and d=0.9R (see blue arrow).

Finally, Supplementary Figure 5 studies a more realistic model for a STML nanocavity, beyond the freestanding-nanoparticle one considered in the main text. The tip geometry consists of a conical body decorated by a spherical protrusion at its apex. We set the radius of the protrusion to 5 nm. The conical height is tens of microns high, and it is embedded into the perfect matching layers at the boundaries of the simulation volume. This way, we remove the EM radiation generated due to the finite truncation of the tip along the vertical direction. We focus our analysis on two geometric parameters (see the right inset): the cone-protrusion vertical overlapping, weighted by d, and the conical angle, given by θ. The left panel shows far-field spectra for θ=105° and three different d. We can observe that the far-field spectrum obtained for small cone-protrusion overlapping (large d, blue line) is very similar to the freestanding nanoparticle one. However, as d decreases, the overlapping increases and $\mathcal{I}_{f-f}$ develops a large, low-frequency tail that distorts the double-peaked spectrum. This feature can be attributed to

the surface plasmon polaritons propagating along the conical surface away from the protrusion. These are leaky modes, which experience larger radiative losses as $h\nu$ decreases.

The inset in the left panel of Supplementary Figure 5 displays induced charge and electric field amplitude maps for θ=105° and d=0.9R, illustrating the key role that the vertical overlapping between tip and protrusion plays in the far-field spectrum. In this case, the geometry presents a sharp junction, which gives rise to strong fields in this position, together with large scattering intensity. This translates into an efficient out-coupling of the gap plasmons supported by the spherical protrusion into the far-field, and prevents the excitation of conical surface plasmon polaritons in the tip. The right panel of Supplementary Figure 5 reveals the robustness of $\mathcal{I}_{f-f}$ against variations in the conical angle for d=0.9R. It shows that variations of the order of 30% in θ barely affects the far-field profiles, which in the three cases considered, resemble the freestanding nanoparticle one. Thus, we can confirm that the model in the main text reproduces accurately the far-field characteristics of STML nanocavities formed by STM tips presenting a well-defined nanometric protrusion at their apex.

Supplementary Note 4: Discussion on the smoothness of the quantum cut-off

For the low voltage regime in the raw plasmonic luminescence spectra, it is interesting to notice that the quantum cut-off is relatively smooth. The light intensity is significantly reduced at photon energies of several hundred meVs below $h\nu_{co}$ in spite of the fact that the broadening of the Fermi level at 4.5 K is only of about 0.3 meV. This effect is easily recognized by comparing the light spectra recorded with a bias voltage of 2.6 V with that recorded at higher bias voltages in Figure 1b: Whereas the main contribution to the emission spectra for higher voltages is the previously mentioned peak at about 2.53 eV, the intensity of this peak is strongly suppressed for V_{bias}^{st}= 2.6 V even though the cut-off is 70 meV above the peak energy.

Normalization makes the transition to the quantum cut-off much sharper (see Figure 5b). For example, the normalized spectra recorded with a voltage of 2.6 V is essentially

indistinguishable from those recorded at higher bias voltages up to photon energies of 2.59 V. Our analysis gives a simple explanation for the smoothness of the quantum cut-off in raw spectra: the light intensity falls to zero when the photon energy approaches the bias voltage at exactly the same rate at which the tunnel intensity vanishes as the voltage approaches zero (see Figure 4). Upon normalization, this smoothness is removed, and the light spectra are comparable up to photon energies which are only separated from the quantum cut-off condition by 10 meV.

Supplementary Note 5: Discussion on the high-bias suppression

While the light intensities in the raw spectra fall by an order of magnitude for stabilization voltages between 3.8 and 4.1 V (see Figure 1d), after normalization the intensity not only does not fall but if anything even shows a slight increase (Figure 5a and b). Thus, the strong decrease in intensity above the high-bias cut-off in the raw spectra can be interpreted as arising from a purely electronic effect, unrelated with the optical coupling between the tip and the sample. Actually, such a decrease in the raw intensities can be traced back to the fact that if the electronic DOS of the sample increases strongly for a given energy E, the elastic current will be affected at bias voltages $eV_{bias} = E$, but the inelastic part will be affected only at a higher bias voltage $eV'_{bias} = E + h\nu$. Since the vast majority of tunneling processes are elastic, under closed feedback conditions, the tip will retract at a bias voltage $eV_{bias} = E$, thereby decreasing the overlap between the initial and final states of the inelastic transitions. This results in a decrease of the inelastic current and, thus, a decrease of the emitted light intensity.

Supplementary Note 6: Discussion on the spectral weight shift.

The shift in the spectral weight and the modification in the intensity ratios between different plasmonic contributions observed in the raw spectra for intermediate voltages can also be understood within the framework developed in this paper. Because the $I(V)$ curve is a

monotonically increasing function of the voltage, the rate of inelastic processes is a monotonically decreasing function of the photon energy (see Equation 4 and Figure 4). Thus, the weight of the different plasmonic contributions decrease with increasing photon energies up to the quantum cut-off, i.e. the light intensity of the plasmonic resonances with energies close to the quantum cut-off will be lower than that of resonances with lower energy. Changing the stabilization bias under closed feedback loop conditions, the quantum cut-off will shift upwards in photon energy, but the slope will decrease since $I\left(V_{bias}^{st}\right) = I_t^{st}$ is fixed under closed-feedback conditions, and thus the tip-surface distance will change so that $\mathcal{R}_{inel}\left(h\nu = 0, V_{bias}^{st}\right)$ also remains constant (See Figure 4). Thus, the intensity of the high energy peaks increases faster with the stabilization bias voltage than the intensity of the low energy peaks. Upon normalization, such dependence is eliminated.

Supplementary Note 7: Different tips, negative voltages and the role of the electronic surface state at Ag(111)

The normalization procedure described in this paper is found to work for any tip and voltage range explored. In particular, when using negative voltages, the conductivity onset at -45 meV related to the surface state (SS) of Ag(111) (Supplementary Figure 6a) should play a role in determining far-field intensities. The generality and accuracy of the method for a different tip with negative voltages as well as the role of the SS is demonstrated in Supplementary Figure 6. The far-field intensities in Supplementary Figure 6b show a marked dependence on the stabilization voltage, similar to the case for positive voltages reported in Figure 1 of the main text. The tip used in the experiments of Supplementary Figure 6b showed to a substantially decreased luminescence intensity, requiring large tunnelling current for its observation (100 nA). Tip-surface distances, thus, are much smaller than the ones shown in the main text, and, as discussed above, we do not expect EM calculations to reproduce their behaviour. The shape of the spectra is indeed quite different from those reported in the main text, with a broad

luminescence band from 1.6 to 2.2 eV and two much narrower peaks (width~50 meV) at 2 and 2.08 eV which appear of similar intensity in the spectra recorded at V_{bias}^{st}=2.7 V (blue solid line), but very different at V_{bias}^{st}=2.1 V (red solid line). The rates of inelastic transitions obtained from the $I(V)$ characteristics are also shown in Supplementary Figure 6b as dashed lines. While the one corresponding to the higher bias voltage displays a rather smooth behaviour, $\mathcal{R}_{inel}(h\nu, V_{bias} = 2.1\text{ V})$ shows a clear kink corresponding to the conductivity onset of the Ag(111) SS. Notice that the far-field light intensity of the peaks at 2 and 2.08 eV are in good agreement with the inelastic rates at these photon energies. Actually, when the far-field intensities are normalized to the inelastic rates as described in Equation (4) of the main text, the spectra recorded at both bias voltages become indistinguishable all the way up to the quantum cut-off, and the intensities of both sharp peaks in the spectrum recorded at the lower bias voltage become also very similar.

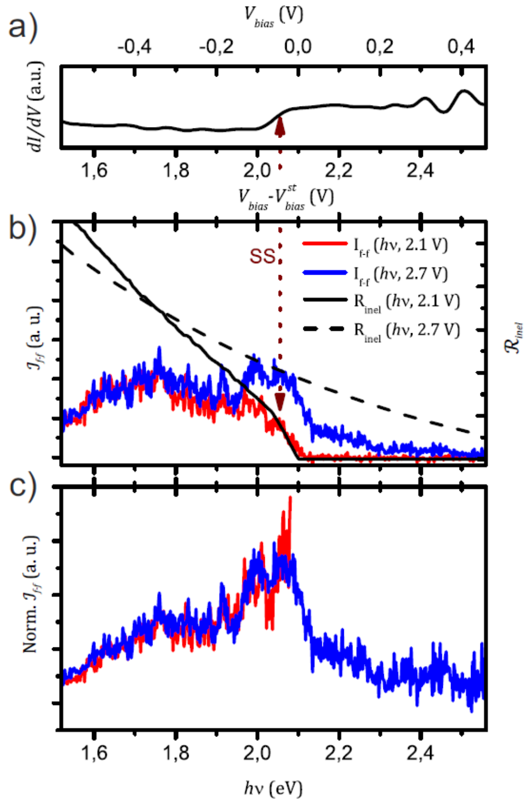

Supplementary Figure 6. a) dI/dV spectra obtained from numerical differentiation of the I(V) curve recorded at a stabilization voltage of 2.1 V. b) Far field light intensities for stabilization voltages of 2.7 V (blue line) and 2.1 V (red line), along with the inelastic rates (solid line for 2.1 V; dashed line for 2.7 V) obtained from the measured $I(V)$ curves. c) Normalized spectra.

Supplementary References

1. Olesen, L., Brandbyge, M., Sørensen, M. R., Jacobsen, K. W., Lægsgaard, E., Stensgaard, I. & Besenbacher. Apparent Barrier Height in Scanning Tunneling Microscopy Revisited. *Phys. Rev. Lett.* **76**, 1485-1488 (1996)

2. Liu, M., Lee, T.-W., Gray, S. K., Guyot-Sionnest, P. & Pelton, M. Excitation of dark plasmons in metal nanoparticles by a localized emitter. Phys. Rev. Lett. 102, 107401 (2009).

3. Ciraci, C., Hill, R. T., Mock, J. J., Urzhumov, Y., Fernández-Domínguez, A. I., Maier, S. A., Pendry, J. B., Chilkoti, A. & Smith, D. R. Probing the Ultimate Limits of Plasmonic Enhancement. *Science* **337**, 1072-1074 (2012).

4. Savage, K. J., Hawkeye, M. M., Esteban, R., Borisov, A. G., Aizpurua, J. & Baumberg, J. J. Revealing the quantum regime in tunnelling plasmonics. *Nature* **491**, 574-577 (2012).
